# Editorial: Pediatric autoimmune neuropsychiatric syndrome

**DOI:** 10.3389/fnbeh.2024.1467469

**Published:** 2024-08-06

**Authors:** Natashia Bottoms, Sydney Rice, Aravindhan Veerapandiyan

**Affiliations:** ^1^Arkansas Children's Hospital, University of Arkansas for Medical Sciences, Little Rock, AR, United States; ^2^University of Arizona Health Sciences, Tucson, AZ, United States

**Keywords:** IVIg, tics, obsessive-compulsive disorder (OCD), food restriction, Pediatric Autoimmune Neuropsychiatric Syndrome (PANS), Pediatric Autoimmune Neuropsychiatric Disorder Associated with Streptococcal Infection (PANDAS)

Pediatric Autoimmune Neuropsychiatric Disorder Associated with Streptococcal Infection (PANDAS) and its related condition, Pediatric Autoimmune Neuropsychiatric Syndrome (PANS), are recognized as autoimmune neurological disorders (Chang et al., 2015). They are thought to result from molecular memory and disruption of the blood-brain barrier due to infections, metabolic issues, or other inflammatory processes. PANDAS specifically involves infections with group A beta-hemolytic streptococci (GAS), mirroring the mechanism seen in Sydenham's chorea. It is characterized by five clinical criteria: the presence of either OCD or tic disorders, onset before puberty, acute symptom onset with a relapsing-remitting course, neurological abnormalities, and a link to GAS. In contrast, PANS serves as a broader term encompassing a range of neurological and psychiatric symptoms, such as OCD, food restriction, tics or choreiform movements, separation anxiety, academic challenges, sleep disturbances, urinary issues, sensory defensiveness, and inattention or hyperactivity arising in the setting of a variety of inflammatory conditions. These symptoms are sudden onset and follow a relapsing-remitting pattern, often responding well to immunomodulatory treatments (Allen et al., 1995; Chang et al., 2015; Swedo et al., 2015).

Diagnosing PANS and PANDAS is challenging due to the lack of reliable biomarkers, relying entirely on clinical criteria. While social media has increased awareness of rare diseases like PANS and PANDAS, numerous studies indicate that these platforms also facilitate the spread of false or misleading health information (Suarez-Lledo and Alvarez-Galvez, 2021). Additionally, clinical management of PANS and PANDAS is complicated by a shortage of rigorous, high-quality studies that define best practices for diagnosis and treatment. This can result in overly broad application of clinical criteria or inappropriate therapies being prescribed due to parental pressure and clinicians' uncertainty about these disorders. In this Research Topic, we present four scientifically rigorous, peer-reviewed studies that explore the incidence, workup, and treatment of PANS/PANDAS, aiming to enhance clinical proficiency and promote best practices.

The first study by Wald et al. presents a multi-center retrospective review examining the incidence of PANS/PANDAS in primary care populations across three geographically and demographically diverse academic medical centers. The findings revealed unexpected variations in incidence rates based on location, which were correlated with racial disparities and types of insurance. Potential cases were identified by reviewing data for one of five qualifying diagnoses (notably excluding tic/movement disorders), and cases were subsequently manually assessed for adherence to standardized PANS/PANDAS clinical criteria. Out of 95,498 patients, 357 were identified as potential cases, with 13 confirmed, resulting in an annual incidence rate of 1 in 11,765. Interestingly, one academic center reported no confirmed cases, leading researchers to speculate that certain “pandogenic” strains might vary by geographic location. However, this center also had a significantly higher racial diversity and a greater proportion of children on public health insurance. The researchers suggest that this discrepancy could be due to diagnostic biases toward patients or parents who raise concerns after encountering information online.

In terms of laboratory workup, Leonardi et al. conducted a cross-sectional study involving 26 patients with PANDAS and 11 controls with recurrent pharyngotonsillitis. This study aimed to perform extensive immunological assessments to identify potential deficits or dysregulation in immune markers, supporting the hypothesis of a pathogenic systemic inflammatory state in PANDAS. Children with PANDAS exhibited higher levels of tumor necrosis factor-alpha and interleukin-17, both of which have been hypothesized to play a role in blood-brain barrier permeability (Takata et al., 2021). However, these differences did not reach statistical significance, likely due to the rarity of the condition resulting small sample size and reduced statistical power. Notably, ASO levels were elevated in nearly all subjects, highlighting the test's unreliability in distinguishing PANDAS from other clinical conditions. The study found no inborn errors of immunity in either group. This indicates that routine screening for immune deficiencies may not be needed in patients with PANS/PANDAS unless additional symptoms of immunodeficiency are present. However, the data did reveal correlations between PANDAS and maternal autoimmune disorders, particularly maternal autoimmune thyroiditis, as well as a higher prevalence in male patients compared to controls.

Pooni et al. analyzed cerebrospinal fluid (CSF) from 471 patients who underwent lumbar puncture due to neuropsychiatric deterioration. They found that at least one CSF abnormality was observed in 29% of patients with PANS compared to 40% of patients with autoimmune encephalitis and 45% of patients with “other neuropsychiatric deterioration,” not meeting criteria for either condition. The most common CSF abnormalities across all groups included elevated CSF protein and/or albumin quotient, while elevated IgG index and IgG oligoclonal bands were rare in all three groups.

Finally, Eremija et al. report on a 5-year retrospective study that utilized standardized neuropsychological assessments to evaluate treatment responses to intravenous immunoglobulin in children with PANS. Twelve children, all of whom had not responded to at least one other immunomodulatory treatment including antibiotics, nonsteroidal anti-inflammatory agents, and steroids, underwent a battery of neuropsychological testing before and after receiving intravenous immunoglobulin. Most patients tolerated the treatment well, with only one needing to discontinue due to aseptic meningitis. Eleven out of 12 patients showed improvement in at least one subdomain in their neuropsychological testing. The most common areas of improvement included memory (58%), sensory-motor skills (37%), and visual-motor integration (30%). Notably, unlike the findings of Leonardi et al., five out of the 12 patients were later diagnosed with hypogammaglobulinemia, which required long-term intravenous immunoglobulin treatment. This suggests that there may be higher rates of initial treatment non-response in children with humoral immunodeficiency.

All the studies included in this Research Topic highlighted small sample sizes, rarity of condition, and the absence of consistent, and standardized assessment measures as notable challenges and limitations. Given the profound functional impairment and psychosocial distress faced by these patients and their families, there is an urgent need for further research to refine identification methods, treatment protocols, and educational initiatives for both healthcare professionals and the patients and families regarding these disorders. We hope these papers will not only provide a foundation of scientifically rigorous information but also inspire future academic exploration and discussion in this important field.

During the preparation of this work the author(s) used ChatGPT in order to improve readability and language. After using this tool/service, the author(s) reviewed and edited the content as needed and take full responsibility for the content of the publication.

## Author contributions

NB: Data curation, Writing – original draft, Writing – review & editing. SR: Writing – review & editing. AV: Conceptualization, Data curation, Writing – review & editing.
